# Shedding light on venoarterial PCO_2_ gradient

**DOI:** 10.1186/s13613-017-0266-5

**Published:** 2017-04-11

**Authors:** Arnaldo Dubin, Mario Omar Pozo

**Affiliations:** Cátedra de Farmacología Aplicada, Facultad de Ciencias Médicas, Universidad Nacional de La Plata, 60 y 120, calle 42 No 577, 1900 La Plata, Argentina

As an expression on Fick’s principle, the reduction in cardiac output is associated with a parallel increase in both mixed venoarterial CO_2_ content difference (C_mv-a_CO_2_) and arterial-mixed venous oxygen content difference (C_a-mv_O_2_). Nevertheless, disproportioned elevations in C_mv-a_CO_2_ compared to those of C_a-mv_O_2_ ensue when the anaerobic threshold is reached. This results from anaerobic CO_2_ production, secondary to the buffering of anaerobically generated protons by bicarbonate.

The clinical approach to venoarterial CO_2_ differences usually relies on partial pressure rather than content difference. Unfortunately, the attempts to track C_mv-a_CO_2_ through mixed venoarterial PCO_2_ difference (P_mv-a_CO_2_) might be misleading. The relationship between CO_2_ content and partial pressure is intricate. Moreover, the estimation of CO_2_ content from PCO_2_ is troublesome, and calculation algorithms frequently produce unreliable results. Since several factors can modify the dissociation of CO_2_ from Hb, P_mv-a_CO_2_ can fail to reflect C_mv-a_CO_2_ changes. For example, hemodilution induces opposite changes in P_mv-a_CO_2_ and C_mv-a_CO_2_. The high cardiac output that develops in such situation increases P_mv-a_CO_2_ and reduces C_mv-a_CO_2_ [1]. Other factors, such as metabolic acidosis and Haldane effect, can also play a major role in this relationship and have strong effects on P_mv-a_CO_2_, regardless of cardiac output changes [2].

Another focus of confusion might reside in the actual meaning of venoarterial PCO_2_ difference. Differently to tissue-arterial PCO_2_ difference, P_mv-a_CO_2_ primarily reflects the changes in systemic blood flow and not in microcirculatory perfusion. Physiologic research helps to understand this question. In an experimental model of endotoxemia, all the PCO_2_ differences—P_mv-a_CO_2_, mesenteric venoarterial and mucosal villi-arterial—increased during the phase of hypodynamic shock [3]. After fluid resuscitation, P_mv-a_CO_2_ and mesenteric venoarterial PCO_2_ difference normalized following the improvement in cardiac output and superior mesenteric artery blood flow. Tissue hypercarbia, however, remained present as an expression of villi microcirculatory hypoperfusion.

Although mixed venous and central venous gases are not interchangeable [4], central venoarterial PCO_2_ difference (P_vc-a_CO_2_) has been used a surrogate for P_mv-a_CO_2_. It might thus be a good marker of cardiac output, even more sensitive than central venous oxygen saturation [5]. Nevertheless, an observational study found that P_vc-a_CO_2_ did not correlate with cardiac output but with sublingual microvascular perfusion [6]. It was therefore claimed by some authors that P_vc-a_CO_2_ might reflect tissue perfusion. This speculation is supported neither by physiology [3] nor by relevant clinical studies. In septic shock, patients with a hyperdynamic profile showed lower P_vc-a_CO_2_ than those with normal systemic hemodynamics, even though the microcirculatory alterations were similar in both groups [7]. So, the lack of correlation between cardiac output and P_vc-a_CO_2_ found in septic patients [6] should be explained by modifications in the dissociation of CO_2_ from Hb. Disorders such as hemodilution and lactic acidosis are commonly present in septic shock and frequently display microvascular abnormalities. Certainly, the relationship between P_vc-a_CO_2_ and microcirculation should not be interpreted as a causal phenomenon.

In this issue of *Annals of Intensive Care*, Mallat et al. [8] report that an acute reduction in arterial PCO_2_ from 44 to 34 mm Hg was associated with an increase of 2 mm Hg in P_vc-a_CO_2_. The authors attributed this finding to the concomitant increase in oxygen consumption (VO_2_). Unfortunately, methodological issues might limit the relevance of the conclusions: First, the increase in P_cv-a_CO_2_ not only was quantitatively minor and insignificant from a clinical point of view, but mainly stayed within the error of the method of PCO_2_ measurement. This is especially true when taking into account the error propagation produced during the calculation of the PCO_2_ difference. Furthermore, the use of central venous instead of mixed venous gases for computation of VO_2_ is questionable [4]. In addition, the subtle change in base excess that appeared during hyperventilation might also explain part of the change in P_cv-a_CO_2_ [2]. Modifications in Hb levels before and after hyperventilation, which might have affected P_cv-a_CO_2_ [1], were not reported. A comprehensive discussion about any P_cv-a_CO_2_ change should consider all its determinants.

The effects of hypocapnia on P_vc-a_CO_2_ have been previously reported in stable cardiac surgery patients [9]. An experimental study also showed that severe hypocapnia increased gut intramucosal-arterial PCO_2_ as a probable consequence of regional and tissue hypoperfusion. In contrast, systemic and regional venoarterial PCO_2_ gradients did not change [10]. In this way, the effects of hypocapnia on P_vc-a_CO_2_ are uncertain.

Although the study from Mallat et al. [8] does not add new physiologic information and has major limitations, it emphasizes that P_vc-a_CO_2_ is not a straightforward surrogate for blood flow. The messages for physiologists and practitioners should be that P_vc-a_CO_2_ monitoring might contribute to the assessment of systemic hemodynamics but requires a comprehensive interpretation.

## Abbreviations

C_mv-a_CO_2_: mixed venoarterial CO_2_ content difference
C_a-mv_O_2_: arterial-mixed venous oxygen content difference
P_mv-a_CO_2_: mixed venoarterial PCO_2_ difference
P_vc-a_CO_2_: central venoarterial PCO_2_ difference
VO_2_: oxygen consumption
